# Natural history of 15 patients with autosomal dominant *WFS1* pathogenic variants associated with sensorineural hearing loss and optic atrophy

**DOI:** 10.1186/s13023-026-04348-9

**Published:** 2026-04-18

**Authors:** Jessica P. Roberts, Abby F. Tang, Daniela Hernandez, Brianna Carman, Liam Oiknine, Cris Brown, Stacy Hurst, Yunshuo Tang, Fumihiko Urano

**Affiliations:** 1 Division of Endocrinology, Department of Medicine, Metabolism, and Lipid Research, Washington University School of Medicine, 660 South Euclid Avenue, St. Louis, MO 63110 USA; 2 Department of Pathology and Immunology, Washington University School of Medicine, 660 South Euclid Avenue, St. Louis, MO 63110 USA; 3 Georgetown University School of Medicine, Washington, DC USA; 4 Department of Neurology, Washington University School of Medicine, 660 South Euclid Avenue, St. Louis, MO 63110 USA; 5 Department of Ophthalmology & Visual Sciences, Washington University School of Medicine, 660 South Euclid Avenue, St. Louis, MO 63110 USA

## Abstract

**Objective:**

Autosomal dominant pathogenic variants in *WFS1* cause a spectrum of disorders with phenotypic manifestations including low-frequency sensorineural hearing loss, optic nerve atrophy accompanied by low- to mid-frequency sensorineural hearing loss, non-syndromic diabetes mellitus, and early-onset cataracts. These conditions are generally milder than autosomal recessive Wolfram syndrome, except for Hattersley-Urano syndrome. Despite growing recognition of these disorders, data on clinical progression and long-term outcomes remain limited. This study aims to expand knowledge on disease severity and progression in autosomal dominant *WFS1*-related disorders.

**Approach:**

Clinical data were obtained from the Washington University International Registry and Clinical Study for Wolfram Syndrome and related disorders and the Endoplasmic Reticulum Disease Patient Registry and Biorepository. Fifteen participants with autosomal dominant WFS1 pathogenic variants presenting with both optic nerve atrophy and sensorineural hearing loss were included.

**Results:**

The 15 cases included seven distinct autosomal dominant *WFS1* variants: c.923C>G (p.Ser308Cys), c.2051C>T (p.Ala684Val), c.2389G>T (p.Asp797Tyr), c.2402A>G (p.Asp801Gly), c.2456A>C (p.Gln819Pro), c.2492G>C (p.Gly831Ala), and c.2590G>A (p.Glu864Lys). Among these, the c.2402A>G, c.2456A>C, and c.2492G>C variants have not been previously published. Median age at optic atrophy diagnosis was 10 years. Visual acuity and RNFL thickness did not change significantly with age. Of eight patients with Optical coherence tomography (OCT) scans, six showed outer plexiform layer (OPL) lamination, a characteristic feature of this disorder. Hearing loss was diagnosed at a median age of 2.0 years; all 15 participants use hearing aids, and eight have bilateral cochlear implants.

**Conclusion:**

Patients with autosomal dominant *WFS1*-related disorders experience early-onset hearing loss and late-childhood optic atrophy, with stable visual acuity and RNFL thickness over time. OPL lamination is a highly characteristic finding, and three novel variants with four clinical presentations are described.

## Introduction

Wolfram syndrome 1 (*WFS1*) gene was originally identified in 1998 as the causative gene for Wolfram syndrome, a rare autosomal recessive genetic disorder characterized by antibody-negative early-onset diabetes mellitus, arginine vasopressin deficiency (also known as central diabetes insipidus), optic nerve atrophy, and sensorineural hearing loss, along with other neurological and psychological features [1–6]. Over the past decade, disorders associated with dominant variants of the *WFS1* gene have received increasing attention. These conditions, often referred to as Wolfram-like disorders, have become more diverse and, generally, are less clinically severe compared to Wolfram syndrome. As a result, the broader term “Autosomal dominant *WFS1*-related disorders” is now commonly used. The clinical features associated with Autosomal dominant *WFS1*-related disorders can vary widely. These features include isolated low-frequency sensorineural hearing loss, optic nerve atrophy accompanied by low to mid-frequency sensorineural hearing loss, isolated diabetes mellitus, and isolated early-onset cataracts. While most autosomal dominant *WFS1*-related disorders are generally milder than Wolfram syndrome, there is a severe condition known as Hattersley-Urano syndrome [7]. This syndrome, caused by autosomal dominant *WFS1* variants, is characterized by neonatal diabetes, congenital sensorineural deafness, congenital cataracts, hypotonia, developmental delay, and intellectual disability [7].

Although the natural history of Wolfram syndrome has been extensively studied, the natural history of autosomal dominant *WFS1*-related disorders has received limited research attention. One specific constellation of clinical features in autosomal dominant *WFS1*-related disorders includes optic nerve atrophy and sensorineural hearing loss, which is positioned on the less severe end of the spectrum of disorders caused by *WFS1* variants [8–11]. In this study, we present the clinical features of 15 patients with autosomal dominant *WFS1* variants. Specifically, we focus on metrics of patients’ visual and hearing health. Thus, we have included visual acuity and retinal nerve fiber layer (RNFL) thickness measures, characterization of abnormal retinal structures, and hearing aid use. These findings will add to a small but growing body of literature on the visual and hearing impairments associated with autosomal dominant *WFS1*-related disorder [8–13].

## Materials and methods

### Patient clinical information and genetic analysis

Patient clinical information was obtained from the Washington University International Registry and Clinical Study for Wolfram Syndrome and the Endoplasmic Reticulum Disease Patient Registry and Biorepository. The following are the minimum diagnostic inclusion criteria: [1] Genetic analysis confirmation of an autosomal dominant variant in the *WFS1* gene [2]. Formal diagnosis of optic atrophy and sensorineural hearing loss [3]. Subjects, or their parent or legal guardian, provided signed written, informed consent for participation in the study and release of personal health information prior to their inclusion in this study. This investigation was approved by the Human Research Protection Office at Washington University School of Medicine in St. Louis, MO (IRB IDs #201107067 and #201807044).

### Clinical assessment and data collection

Clinical information on the 15 participants was gathered from the Washington University International Registry and Clinical Study for Wolfram Syndrome, Endoplasmic Reticulum Disease Patient Registry and Biorepository, and patients’ medical records. Collected records include age, sex, *WFS1* variant, age at optic atrophy diagnosis, age at hearing loss diagnosis, hearing aid use, age at hearing aid installation, other vision conditions, other medical conditions, best corrected visual acuity (BCVA) measurements, and RNFL thickness measurements, and optical coherence tomography B-scan images.

### Statistical analyses

We used Proc Mixed of SAS software for windows (V9.4) to implement a repeated measures model to account for two eyes and repeated measurements from the same patient. Proc Mixed of SAS software for Windows (V9.4) was used to test if there was a significant relationship between mean RNFL thickness, BCVA, and age adjusting for multiple observations per participant.

We averaged right and left eye BCVA and RNFL thickness data for each participant at each time point and used R (version 4.5.0) to analyze longitudinal progression of these ophthalmologic measurements. A linear mixed-effects model with age as a fixed effect and subject-specific random intercepts was applied to both datasets. The model was fit using the lmer() function from the lme4 package, with p-values for fixed effects derived from the lmerTest package. Model-predicted mean BCVA and mean RNFL thicknesses across the observed age range were obtained using the ggeffects package and visualized using the ggplot2 package.

## Results

### Participant demographics and clinical features

We identified 15 patients in the Washington University International Registry and Clinical Study for Wolfram Syndrome and the Endoplasmic Reticulum Disease Patient Registry and Biorepository with autosomal dominant variants in the *WFS1* gene that have been diagnosed with optic nerve atrophy and sensorineural hearing loss. A summary of the demographics and clinical features of these patients is provided in Table 1.

**Fig. 1:**
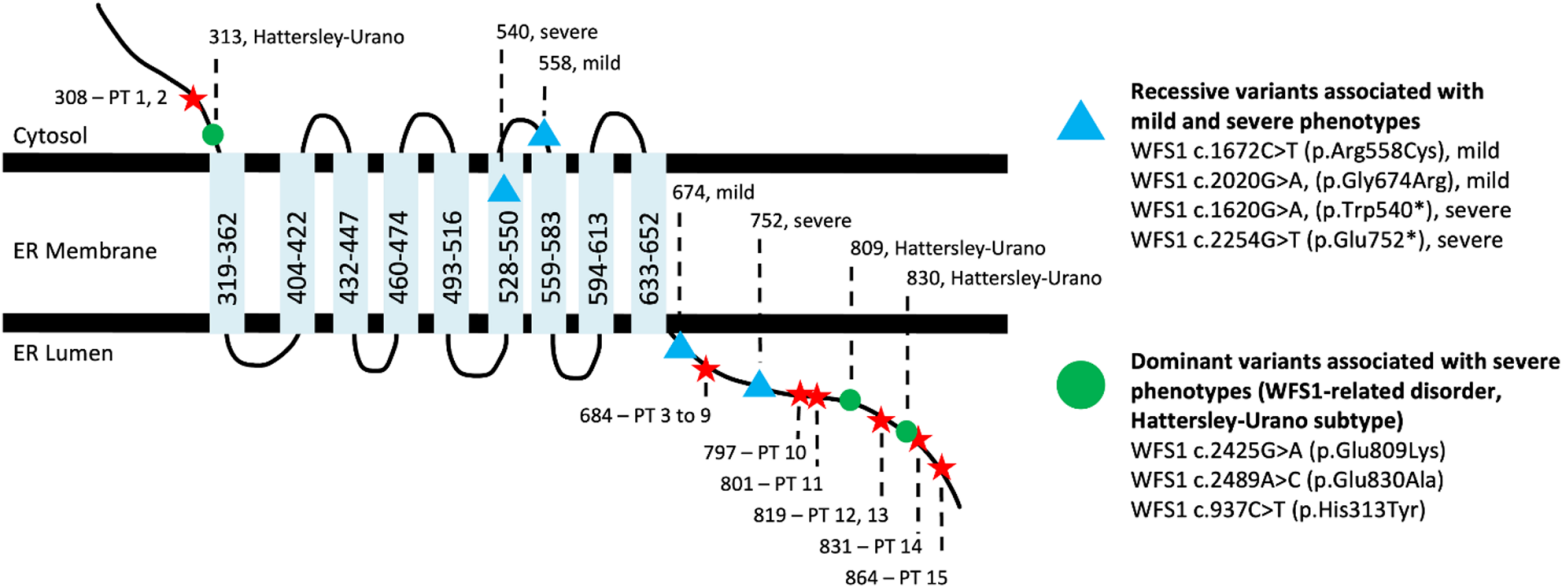
Predicted topological structure of the WFS1 protein, illustrating the positions of the seven autosomal dominant *WFS1* variants identified in this cohort across the cytosolic, transmembrane, and ER luminal domains. Six variants (p.Ala684Val, p.Asp797Tyr, p.Asp801Gly, p.Gln819Pro, p.Gly831Ala, and p.Glu864Lys) are located within the ER luminal domain; one variant (p.Ser308Cys) is predicted to lie within the cytosolic domain. For reference, the approximate positions of variants associated with recessive Wolfram syndrome and more severe autosomal dominant phenotypes (including Hattersley-Urano syndrome) are also shown. ER: endoplasmic reticulum; PT: patient

**Fig. 2:**
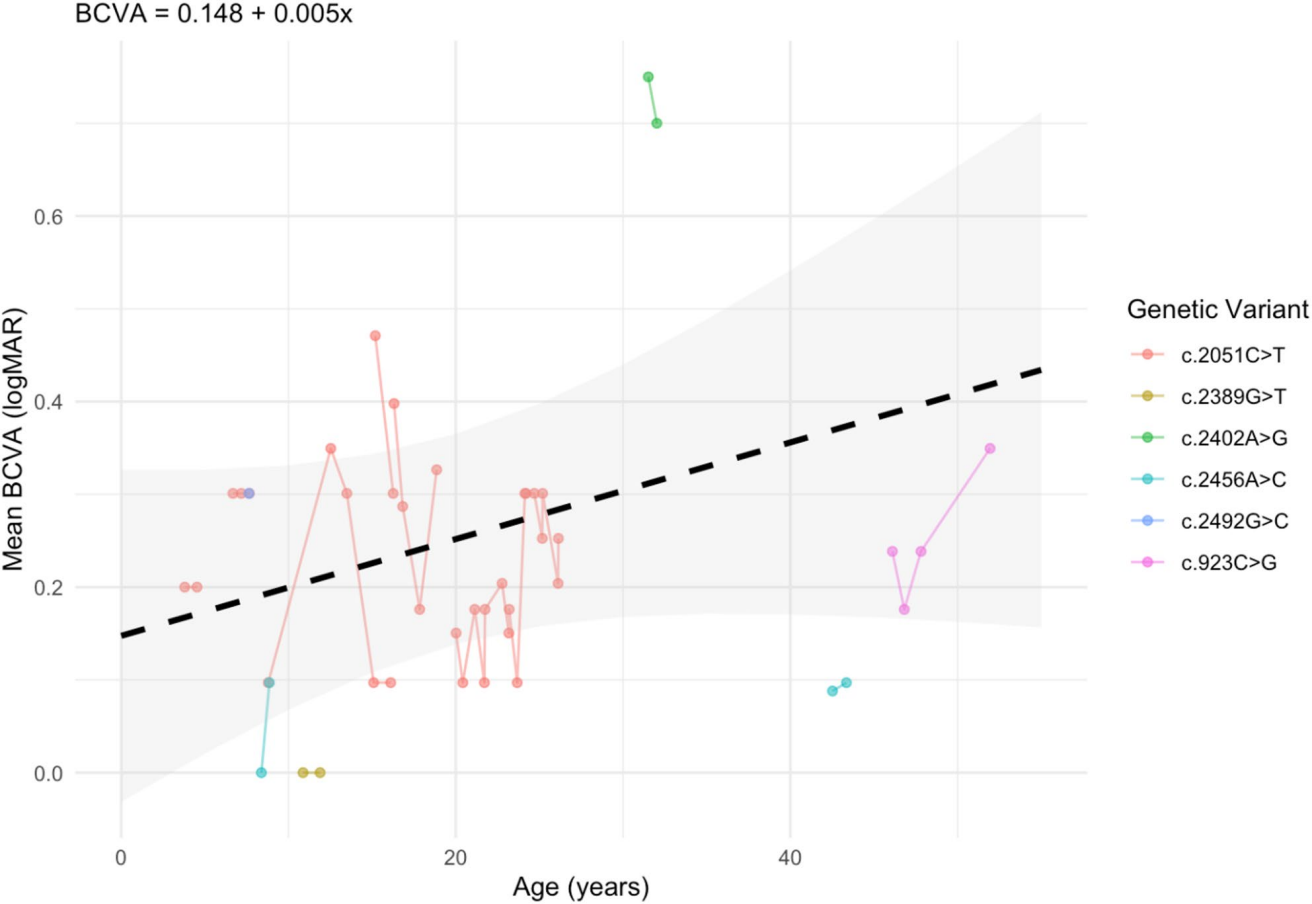
Longitudinal mean (of left and right eye) best-corrected visual acuity (BCVA) of 12 participants. The dashed line represents the estimated slope of 0.05211 logMAR/year (*p* = 0.159) with the grey ribbon representing 95% confidence bands [-0.0017, 0.0121]

**Fig. 3:**
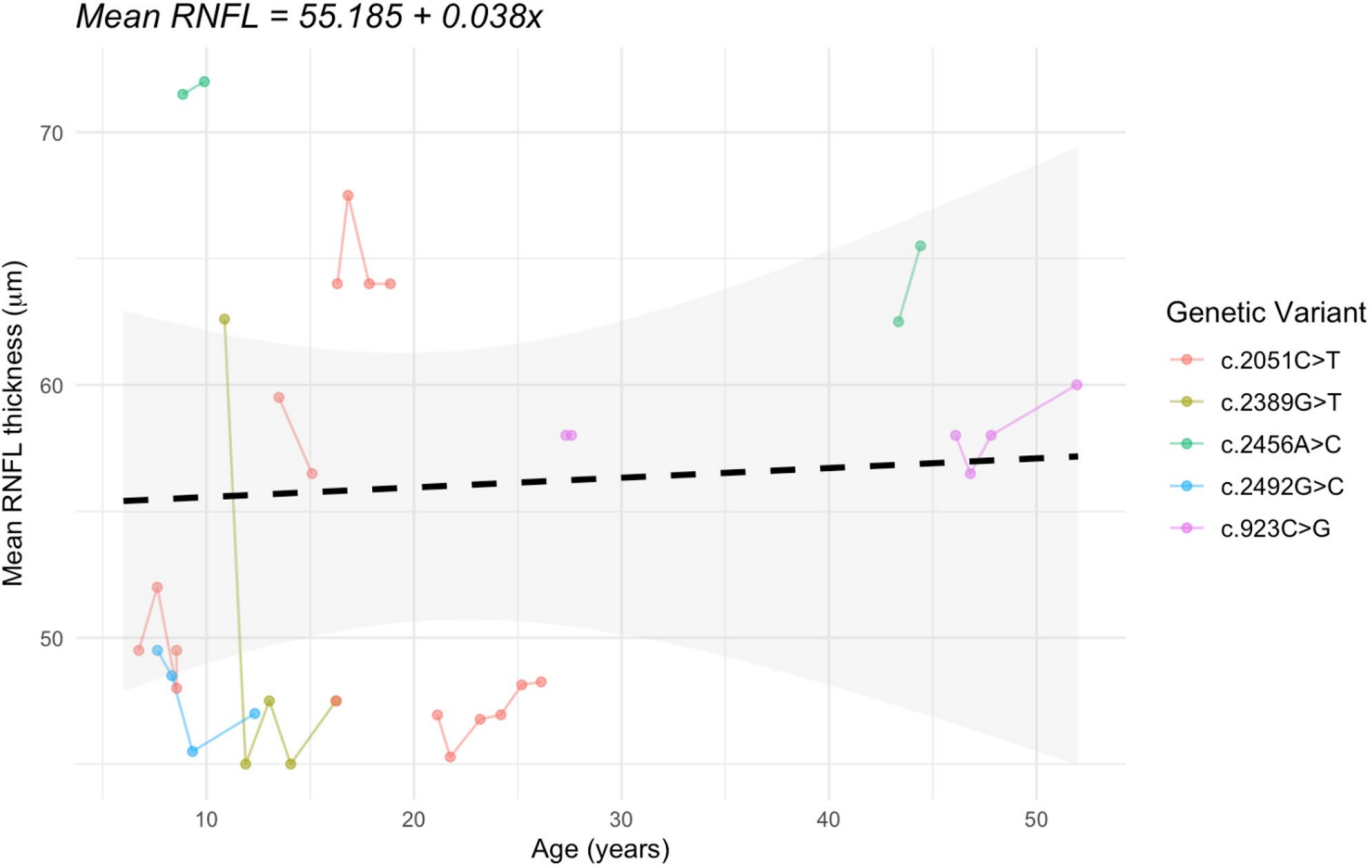
Longitudinal mean (of left and right eye) RNFL thickness of 11 participants. The dashed line represents the estimated slope of 0.03833 μm/year (*p* = 0.829) with the grey ribbon representing 95% confidence bands [-0.3038, 0.3805]

**Fig. 4:**
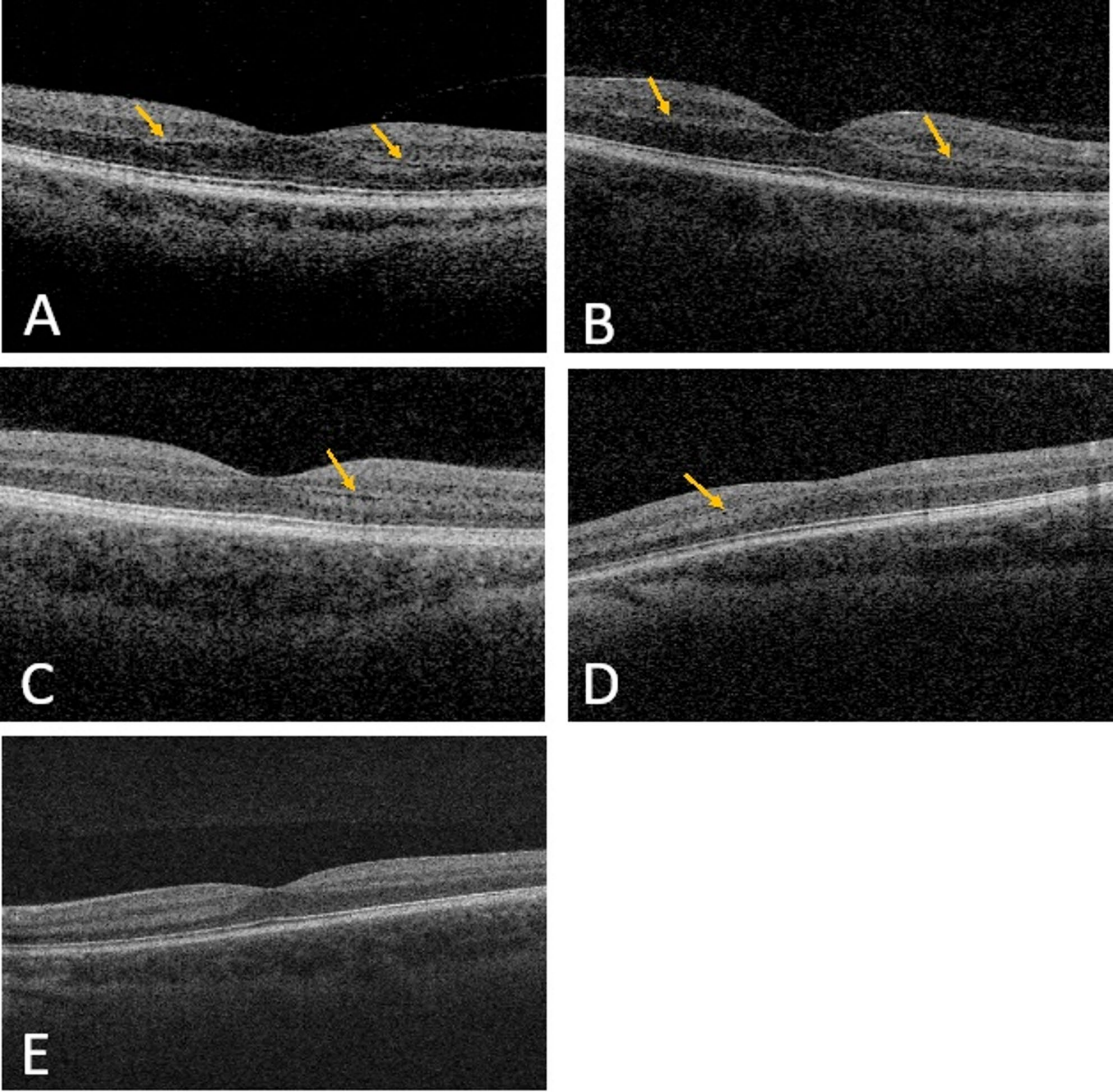
Retinal Outer Plexiform Layer Lamination. **A** through **E** - Optical coherence tomography (OCT) B-scan images of study participants. Patients 1, 2, and 5 exhibit outer plexiform layer (OPL) lamination, as denoted by yellow arrows; patient 13 does not have definitive OPL lamination. **A** - patient 1 (c.923 C > G). **B** - patient 2 (c.923 **C** > **G**). **C** - patient 5 (c.2051 **C** > T). **D** - patient 10 (c.2389G > T). **E** - patient 13 (c.2456 **A** > **C**)

**Table 1:** Clinical and genetic characteristics of the 15 patients with autosomal dominant *WFS1* variants in this cohort

Patient	Sex	Age (years)	Nucleotide variant	Amino acid change	WFS1 Domain	Age at genetic diagnosis (years)	Age at optic atrophy diagnosis (years)	Age at hearing loss diagnosis (years)	Hearing aid use	Age at Hearing Aid Installation (Right | Left)	OPL Lamination	Other Vision Conditions	Other Medical Conditions
1	M	54	c.923 C > G	p.Ser308Cys	Cytosolic	47	42	2	Y - EA	36 y | 36 y	Yes	None	None
2	M	28	c.923 C > G	p.Ser308Cys	Cytosolic	28	10	3	Y - EA	3 y | 3 y	Yes	Color vision deficiency	Tinnitus, anemia, hyperhydrosis, thermoregulatory dysfunction, anxiety, depression, bulimia
3	M	19	c.2051 C > T	p.Ala684Val	ER lumen	9	8	1.5	Y - CI	25 mo | 34 mo	Yes	Myopia	Achille’s tendon contractures, toe-walking
4	M	27	c.2051 C > T	p.Ala684Val	ER lumen	20	19	2	Y	NA	NA	Myopia, astigmatism, reduced color perception	Diabetes mellitus, thoracolumbar scoliosis
5	F	20	c.2051 C > T	p.Ala684Val	ER lumen	15	15	2	Y - CI	13 y | 12 y	Yes	Color blindness	None
6	F	10	c.2051 C > T	p.Ala684Val	ER lumen	4	6	2	Y - CI	4 y |5 y	NA	None	None
7	M	6	c.2051 C > T	p.Ala684Val	ER lumen	3	3	0.3	Y - CI	3 y | 3 y	NA	None	Developmental speech delay
8	M	6	c.2051 C > T	p.Ala684Val	ER lumen	3	3	0	Y - CI	2 y | 3 y	NA	None	None
9	F	20	c.2051 C > T	p.Ala684Val	ER lumen	16	16	0	Y - CI	2.5 y | 2.7 y	NA	None	Polycystic ovary syndrome, beta thalasemmia trait
10	M	22	c.2389G > T	p.Asp797Tyr	ER lumen	12	10	2	Y - CI	4 y | 8 y	Yes	None	None
11	M	33	c.2402 A > G	p.Asp801Gly	ER lumen	32	25	4.5	Y - CI/EA	NA | 16 y	Yes	Nystagmus	None
12	F	44	c.2456 A > C	p.Gln819Pro	ER lumen	43	42	5	Y - EA	NA	No definitive lamination	Myopia, elevated intraocular pressure, color blindness, peripheral visual field constriction	Chiari I malformation, migraines, hypothyroidism, sleep apnea, endometriosis
13	F	10	c.2456 A > C	p.Gln819Pro	ER lumen	8	8	1.6	Y - EA	4 y | 4 y	No definitive lamination	Myopia, astigmatism	Type 1 diabetes mellitus, bilateral hip dysplasia
14	F	18	c.2492G > C	p.Gly831Ala	ER lumen	7	6	0.5	Y - CI	1 y | 2 y	NA	Color blindness	Celiac disease, eczema, ADHD, disruptive behavior disorder
15	F	43	c.2590 G > A	p.Glu864Lys	ER lumen	39	13	4	Y	NA	NA	Color vision deficiency	Migraines, fibromyalgia, narcolepsy, anxiety, depression, PTSD

ADHD: attention-deficit/hyperactivity disorder; CI: cochlear implant; EA: external hearing aid; ER: endoplasmic reticulum; F: female; M: male; OPL: outer plexiform layer; PTSD: post-traumatic stress disorder

**Table 2:** Genetic testing results for the 15 patients in this cohort. GJB2, GJB3, and GJB6 are connexin-encoding genes in which pathogenic variants cause hereditary hearing loss; sequencing of these genes was performed as part of hearing loss genetic panels in select patients; NGS: next-generation sequencing; VUS: variant of uncertain significance; WES: whole-exome sequencing

Patient	Amino acid change	Variant Classification	Prior publications	Genetic Testing Company	Genetic Testing Panel	Type of Sequencing	GJB2/3/6 Gene Sequencing
1	p.Ser308Cys	VUS	Yes	Prevention Genetics	Targeted Sequencing of *WFS1* gene	NGS	No
2	p.Ser308Cys	VUS	Yes	GeneDx	Combined Mito Genome Plus Mito Nuclear Gene Panel	NGS	No
3	p.Ala684Val	Pathogenic	Yes	University of Iowa: Molecular Otolaryngology and Renal Research Laboratories	Hearing Loss Panel	NGS	Yes
4	p.Ala684Val	Pathogenic	Yes	Mendelics	Hereditary Retinopathies Panel	NGS	No
5	p.Ala684Val	Pathogenic	Yes	Invitae	Inherited Retinal Disorders Panel	NGS	No
6	p.Ala684Val	Pathogenic	Yes	Blueprint Genetics	Comprehensive Hearing Loss and Deafness Panel	NGS	Yes
7	p.Ala684Val	Pathogenic	Yes	Great Ormond Street Hospital for Children	Hearing Loss Panel	NGS	Yes
8	p.Ala684Val	Pathogenic	Yes	Greenwood Genetic Center	Targeted sequencing of *OTOF* gene (negative), then Quick Analysis based on clinical presentation	NGS	Unknown
9	p.Ala684Val	Pathogenic	Yes	Invitae	Inherited Retinal Disorders Panel	NGS	No
10	p.Asp797Tyr	Likely pathogenic	Yes	Prevention Genetics	Optic Atrophy NextGen Sequencing Panel	NGS	No
11	p.Asp801Gly	VUS	No	Invitae	Targeted Sequencing of *WFS1* gene	NGS	No
12	p.Gln819Pro	VUS	No	GeneDx	XomeDxPlus / Clinical Exome Sequence Analysis	NGS	Yes
13	p.Gln819Pro	VUS	No	GeneDx	XomeDxPlus / Clinical Exome Sequence Analysis	NGS	Yes
14	p.Gly831Ala	VUS	No	GeneDx	XomeDx / Whole Exome Sequence Analysis	NGS	Yes
15	p.Glu864Lys	Pathogenic	Yes	GeneDx	XomeDx / Clinical Exome Sequence Analysis	NGS	Yes

The median age of patients in this study is 20.0 years (lower and upper quartiles: 14.0 and 30.5). The median age at optic atrophy diagnosis is 10 years (7.0 and 17.5). Four patients have myopia (4/15), five patients have color vision deficiencies (5/15), two patients have astigmatism (2/15), one patient (1/15) has elevated intraocular pressure and peripheral visual field constriction, and one patient (1/15) has nystagmus.

For sensorineural hearing loss diagnosis, the median age was 2.0 years (1.0 year and 2.5). All participants use hearing aids (15/15); eight (8/15) have bilateral cochlear implants, while four (4/15) use bilateral external hearing aids, one patient has a cochlear implant and external aid (1/15), and the hearing device type of two (2/15) patients is unknown. The median time between hearing loss diagnosis and first use of hearing aids was 2.2 years (1.6 and 4.6).

### *WFS1* genotypes and predicted domain locations

The WFS1 genetic variant of each patient is included in Table 1. Variant pathogenicity classifications were assigned in accordance with the American College of Medical Genetics and Genomics (ACMG) standards and guidelines for the interpretation of sequence variants (Table 2) [14]. Seven missense autosomal dominant *WFS1* variants are presented in this study, including three previously unreported variants: c.2402 A > G (p.Asp801Gly), c.2456 A > C (p.Gln819Pro), and c.2492G > C (p.Gly831Ala). Seven (7/15) patients possess the c.2051 C > T (p.Ala684Val) variant, making it the most prevalent in the cohort. Two patients have a c.923 C > G (p.Ser308Cys) variant, which patient 2 paternally inherited from patient 1. Furthermore, patients 12 and 13 share the c.2456 A > C (p.Gln819Pro) variant, which patient 13 maternally inherited from patient 12. Six (6/7) of the altered amino acids have a predicted location in the endoplasmic reticulum (ER) luminal domain of the *WFS1* protein, whereas the p.Ser308Cys variant is likely positioned within the cytosolic *WFS1* domain [15]. A schematic structure of the WFS1 protein is shown with the positions of the *WFS1* variants discussed in this article (Fig. 1).

### Visual acuity

Quantitative measures of vision, such as best corrected visual acuity (BCVA), have been implemented to chart the progression of vision loss in Autosomal dominant Wolfram-related disorders, with previous reports indicating that dominant *WFS1* variants, similarly to recessive variants, lead to progressive vision loss [8, 12, 16, 17]. We sought to characterize the trajectory of vision loss in this study’s cohort given the inclusion of novel autosomal dominant *WFS1* variants and rarity of the condition.

In this cohort, best corrected visual acuity (BCVA) measurements of 12 participants were available, of which 11 participants had data from two or more time points and 10 participants had eye specific data. There was no overall difference between the BCVA for left and right eyes, *p* = 0.9736. The least square BCVA means for the right and left eyes were 0.2501 ± 0.0255 logMAR and 0.2489 ± 0.0255 logMAR, respectively. Using a repeated measures model, we analyzed the trajectory of right and left visual acuity for patients with longitudinal data. Age was not significantly related to longitudinal BCVA, *p* = 0.3185. The estimated slope was 0.00019 logMAR/year (95% CI [-0.0019, 0.0057]). Additionally, we averaged right and left BCVA values and plotted the trajectory of visual acuity for each patient in Fig. 2. A mixed-measures model of mean BCVA also indicated a statistically insignificant relationship between age and BCVA (*p* = 0.159). The estimated slope was 0.05211 logMAR/year (95% CI [-0.0017, 0.0121]).

### Retinal nerve fiber layer thickness

In addition to visual acuity, measurements of retinal nerve fiber layer (RNFL) thickness using optical coherence tomography (OCT) provides another objective measure of disease progression secondary to *WFS1* variant-mediated pathology [8, 12, 16, 17]. The RNFL thickness measurements of 11 participants were available, of which 10 participants had data for two or more time points. Using a repeated measures model to analyze RNFL thickness, there was no difference between the right and left eyes for RNFL, *p* = 0.6515. The least square RNFL means for the right eye and left eye were 55.0 ± 1.41 μm and 54.0 ± 1.41 μm, respectively. Age was not significantly related to mean RNFL thickness (*p* = 0.1547). The slope estimate for age is 0.1513 μm/year (95% CI [-0.0612, 0.3637]). Participant 13 is an influential observation/outlier in the RNFL analysis. Removing this data point leads to a statistically significant relationship between RNFL and age, *p* = 0.0150. The slope estimate is 0.2285 μm/year (95% CI, 0.049 to 0.4083). Furthermore, right and left eye mean thickness values of each participant were averaged for each time point and plotted longitudinally in Fig. 3. The slope estimate for averaged mean RNFL thickness is 0.03833 μm/year (95% CI [-0.3038 to 0.3805]) and did not have a statistically significant relationship with age (*p* = 0.829).

### Outer plexiform layer lamination

Additionally, prior studies on autosomal dominant *WFS1*-related disorders have reported that patients with autosomal dominant *WFS1* have abnormal reflectivity, or lamination, of the outer plexiform layer (OPL) of the retina [8, 12, 13, 16–19]. Among the eight patients in this study with optical coherence tomography (OCT) scans that clearly visualized the OPL, six (6/8) patients had visible OPL lamination. Representative scans of four patients with OPL lamination and one patient without are depicted in Fig. 4.

## Discussion

Here, we have reported the clinical features of 15 patients with autosomal dominant *WFS1* variants, including three novel variants. Notably, all participants received sensorineural hearing loss diagnoses early in life, at a median age of 2 years, and optic nerve atrophy diagnoses at a median age of 10 years. This timeline of sensorineural hearing loss diagnosis within the first few years of life and optic nerve atrophy diagnosis in the second decade of life is consistent with prior reports of the autosomal dominant *WFS1*-related disorder patient population [8, 20, 21]. This is in contrast to patients with autosomal recessive Wolfram syndrome patients, who initially experience high frequency hearing loss in their second decade with progressive loss of lower frequencies [22]. However, both autosomal recessive and autosomal dominant *WFS1* variants lead to onset of optic atrophy in the early teen years [3, 4, 6].

Our analysis of BCVA in patients with autosomal dominant *WFS1* variants did not indicate that there is a strong relationship between BCVA and age, with individual participants demonstrating various rates of decline in visual acuity. Other studies have also noted variable patterns of decline in visual acuity, with one study noting accelerating visual acuity decline with age [12]. Furthermore, BCVA might not be the ideal marker of optic atrophy progression due to the confounding effect of other visual conditions on this measurement. In our cohort, four patients had additional visual conditions that might alter visual acuity. Thus, we also examined mean RNFL thicknesses.

The mean RNFL thickness for the 11 patients in the cohort ranged from 45 to 72 μm, which is markedly below typical RNFL thicknesses for healthy children and adults [23, 24]. Analyses indicated that there is not a strong correlation between patient age and change in RNFL thickness, which aligns with some prior reports of autosomal dominant *WFS1* disorders [12]. It is possible that other factors, such as *WFS1* genetic variant play a more substantial role in decline in RNFL thickness compared to age. Notably, patients 12 and 13, harboring the novel c.2456 A > C (p.Gln819Pro) variant, have the first and third thickest RNFLs in the cohort at 62.5 and 71.5 μm, respectively. Recruitment of additional patients will help further weigh the impact of *WFS1* variant on clinical progression. Studies on autosomal recessive Wolfram Syndrome have also noted that RNFL thickness progression was correlated less with age and more with disease severity [25]. Thus, it is possible that the c.2456 A > C (p.Gln819Pro) variant is associated with less severe manifestations of Autosomal dominant Wolfram-related disorder. Another factor to consider for the insignificant change in RNFL thickness with age is the RNFL thickness “floor”, which refers to the minimum thickness that optical coherence tomography (OCT) instruments can accurately measure. This instrument limitation may lead to fluctuation of measurements taken for patients in this cohort due to very thin RNFL thickness [26]. Overall, our analyses suggest that patients with Autosomal dominant *WFS1*-related disorders may initially present with reduced RNFL thickness; however, the progression of RNFL thinning is often minimal and appears to correlate more closely with disease severity than chronological age.

In addition to reductions in RNFL thinning, lamination of the retinal OPL has surfaced as a characteristic finding of autosomal dominant *WFS1*-related disorder [8, 12, 13, 16–19]. It was postulated to be a pathognomonic feature of autosomal dominant *WFS1* variants [13]. Although one case of OPL lamination in a patient with Wolfram syndrome carrying compound heterozygous c.2339G > C and c.2452 C > T *WFS1* variants has been reported, this is the only reported case so far [17], while multiple studies have reported lamination in the majority of dominant patients. Thus, OPL lamination could be unique to autosomal dominant *WFS1* variants and be more strongly associated with this pattern of inheritance. Interestingly, patient 12 and 13 are the only patients with available scans who do not have definitive OPL lamination and have thicker RNFLs compared to most of the cohort. This further suggests that the c.2456 A > C (p.Gln819Pro) variant might lead to a milder form of autosomal dominant *WFS1*-related disorder compared to other autosomal dominant variants.

The clinical findings observed in our cohort are driven by underlying molecular mechanisms that underscore the essential role of *WFS1* in maintaining cellular homeostasis and protecting against neurodegeneration. *WFS1* gene encodes wolframin (*WFS1* protein), a transmembrane glycoprotein primarily localized to the endoplasmic reticulum (ER) [27]. While *WFS1* is expressed ubiquitously, its expression is notably higher in neurons and pancreatic β cells [2, 27, 28]. *WFS1* protein plays a central role in regulating cellular calcium homeostasis, particularly facilitating Ca^2+^ transfer from the ER to mitochondria through interactions with neuron calcium sensor 1 (NCS1) [29–31]. In an in-silico model, it has been demonstrated that the p.Ala684Val variant, present in seven of the 15 cases reported here, destabilizes the *WFS1* alpha helix, disrupting its interaction with NCS1 [9]. This disruption decreases NCS1 levels and leads to downstream mitochondrial respiratory chain dysfunction [29, 30]. Additionally, *WFS1* interacts with the inositol 1,4,5-triphosphate receptor (IP3R) Ca^2+^ channel, which is critical for intracellular Ca^2+^ balance [32]. Pathogenic *WFS1* variants can impair this interaction, resulting in Ca^2+^ imbalance and triggering mitophagy, ultimately contributing to cellular degeneration [32]. Beyond its role in calcium homeostasis, *WFS1* also functions as a negative regulator of the unfolded protein response (UPR) by interacting with activating transcription factor 6α (ATF6α) [33]. Thus, loss of function of *WFS1* deregulates ER stress pathways, exacerbating cellular ER stress and dysfunction [28, 33]. The C-terminal domain of *WFS1* binds to the ER-localized Na+/K+ ATPase beta-1 subunit (ATP1B1) and facilitates its localization to the cell surface [34–36]. Six of seven variants in this study (p.Ala684Val, p.Asp797Tyr, p.Asp801Gly, p.Gln819Pro, p.Gly831Ala, and p.Glu864Lys) are positioned within the ER lumen domain, where they may disrupt this interaction. Studies in mice have shown that a homozygous p.Glu864Lys variant results in defective ATP1B1-*WFS1* interactions, leading to impaired endocochlear potential, stria vascularis dysfunction, and neurosensory epithelium abnormalities [36]. It is plausible that the other ER lumen variants could similarly affect ATP1B1 function, contributing to sensorineural hearing loss. These molecular insights provide a framework for understanding the clinical manifestations observed in our cohort, particularly the characteristic patterns of optic atrophy and sensorineural hearing loss.

Our findings advance the understanding of optic atrophy and sensorineural hearing loss progression in patients with autosomal dominant *WFS1*-related disorders. The clinical and genetic heterogeneity observed in this cohort underscores the importance of comprehensive phenotypic and molecular characterization. Although BCVA remained relatively stable over time, baseline RNFL thickness measurements demonstrated marked thinning that did not correlate with chronological age. This suggests that substantial RNFL loss likely occurs prior to diagnosis, reflecting early onset and progression of optic atrophy. The apparent stability in later stages may be confounded by the OCT floor effect, which can obscure ongoing degeneration and give the false impression of disease stabilization. These observations emphasize how delayed diagnosis limits our ability to capture the dynamic phase of neurodegeneration in Wolfram and related disorders.

This study has several limitations. The retrospective design and small cohort size (*n* = 15) limit the statistical power to detect associations between age and visual acuity or RNFL thickness, and introduce variability in BCVA and RNFL measurements across clinical sites. Additionally, four variants in this cohort are classified as variants of uncertain significance (VUS) under ACMG criteria [14]. Therefore, genotype–phenotype correlations for these patients should be interpreted with caution. These are discussed further below. Prospective, multi-site studies with larger cohorts will be needed to better characterize the longitudinal trajectories of visual acuity and RNFL thickness in autosomal dominant *WFS1*-related disorders. Genetic testing reports for all patients are summarized in Table 2.

Furthermore, patients in this study were retrospectively selected from a repository. All patients received Next-Generation Sequencing performed at a laboratory certified by the Clinical Laboratory Improvements Amendment (CLIA) program. However, many patients did not have biological samples available to confirm the genetic findings of prior genetic sequencing for this study. As many patients reside far from our clinical center, it was not feasible to collect biological samples. Additionally, while some patients received multigene panels for hearing loss and exome sequencing, not all patients received comprehensive testing to determine if other variants contribute to their phenotype (Table 2).

Lastly, four variants included in this case series ar” classified as “variants of uncertain significance.“ However, no alternative genetic diagnoses have been identified. Additionally, the genetic database Franklin lists the heterozygous *WFS1* c.923C > G and c.2492G > C variants as likely pathogenic (https://franklin.genoox.com). Further functional, segregation, and population data will help refine the classification of these *WFS1* variants.

This study expands the clinical and genetic characterization of autosomal dominant *WFS1*-related disorders, describing three previously unreported variants and highlighting OPL lamination as a highly characteristic retinal feature. The early onset of both hearing loss and optic atrophy in this cohort, combined with evidence of substantial RNFL thinning prior to diagnosis, underscores the need for prospective longitudinal studies that enroll patients at or before symptom onset. As earlier genetic diagnosis becomes increasingly feasible, there is a growing opportunity to capture the dynamic phase of neurodegeneration that retrospective cohorts inevitably miss. Larger multi-site studies will be essential for establishing genotype–phenotype correlations, refining variant classifications, and ultimately informing the development of targeted therapies for this under-recognized spectrum of disorders.

## Data Availability

The clinical data supporting the findings of this study are not publicly available due to patient privacy considerations and restrictions of the IRB-approved protocols governing the Washington University International Registry and Clinical Study for Wolfram Syndrome (IRB #201107067) and the Endoplasmic Reticulum Disease Patient Registry and Biorepository (IRB #201807044). Deidentified data may be made available from the corresponding author, Fumihiko Urano, MD, PhD (urano@wustl.edu), upon reasonable request and subject to institutional review and execution of an appropriate data use agreement.
